# Supplementary material for: HIVprotI: an integrated web based platform for prediction and design of HIV proteins inhibitors
Source: J Cheminform. 2018 Mar 9;10:12. doi: 10.1186/s13321-018-0266-y (PMC5845081; doi:10.1186/s13321-018-0266-y)
Supplement: Supplementary file 2 — Additional file 2. Source code of HIVProtI web server. [file 13321_2018_266_MOESM2_ESM.zip › HIVprotI_Source-code/hivprotI/weka-3-6-6/doc/weka/classifiers/meta/Decorate.html]

Decorate


---


|  |  |  |
| --- | --- | --- |
| **Overview** \| **Package** \| **Class** \| **Tree** \| **Deprecated** \| **Index** \| **Help** \| **Weka's home** | |  |
| **PREV CLASS**   **NEXT CLASS** | **FRAMES**    **NO FRAMES**     **All Classes** |  |
| SUMMARY: NESTED \| FIELD \| CONSTR \| METHOD | DETAIL: FIELD \| CONSTR \| METHOD |  |


---


## weka.classifiers.meta Class Decorate

```
java.lang.Object
  weka.classifiers.Classifier
      weka.classifiers.SingleClassifierEnhancer
          weka.classifiers.IteratedSingleClassifierEnhancer
              weka.classifiers.RandomizableIteratedSingleClassifierEnhancer
                  weka.classifiers.meta.Decorate
```

**All Implemented Interfaces:**: java.io.Serializable, java.lang.Cloneable, CapabilitiesHandler, OptionHandler, Randomizable, RevisionHandler, TechnicalInformationHandler

---

``` public class Decorate extends RandomizableIteratedSingleClassifierEnhancer implements TechnicalInformationHandler ```

DECORATE is a meta-learner for building diverse ensembles of classifiers by using specially constructed artificial training examples. Comprehensive experiments have demonstrated that this technique is consistently more accurate than the base classifier, Bagging and Random Forests.Decorate also obtains higher accuracy than Boosting on small training sets, and achieves comparable performance on larger training sets.
